# Lower endoscopic delivery of freeze-dried intestinal microbiota results in more rapid and efficient engraftment than oral administration

**DOI:** 10.1038/s41598-021-84152-6

**Published:** 2021-02-25

**Authors:** Christopher Staley, Hossam Halaweish, Carolyn Graiziger, Matthew J. Hamilton, Amanda J. Kabage, Alison L. Galdys, Byron P. Vaughn, Kornpong Vantanasiri, Raj Suryanarayanan, Michael J. Sadowsky, Alexander Khoruts

**Affiliations:** 1 Division of Basic & Translational Research, Department of Surgery, University of Minnesota, Minneapolis, MN USA; 2 BioTechnology Institute, University of Minnesota, Saint Paul, MN USA; 3 Division of Gastroenterology, Department of Medicine, University of Minnesota, 2101 6th St. S.E., Room 3-184, Wallin Biomedical Sciences Building, Minneapolis, MN 55414 USA; 4 Division of Infectious Diseases and International Medicine, Department of Medicine, University of Minnesota, Minneapolis, MN USA; 5 Department of Pharmaceutics, College of Pharmacy, University of Minnesota, Minneapolis, MN USA; 6 Department of Soil, Water, and Climate, University of Minnesota, Saint Paul, MN USA; 7 Department of Plant and Microbial Biology, University of Minnesota, Saint Paul, MN USA

**Keywords:** Computational biology and bioinformatics, Drug discovery, Microbiology, Diseases, Gastroenterology, Medical research

## Abstract

Fecal microbiota transplantation (FMT) is a highly effective treatment for recurrent *Clostridioides difficile* infection (rCDI). However, standardization of FMT products is essential for its broad implementation into clinical practice. We have developed an oral preparation of freeze-dried, encapsulated microbiota, which is ~ 80% clinically effective, but results in delayed engraftment of donor bacteria relative to administration via colonoscopy. Our objective was to measure the engraftment potential of freeze-dried microbiota without the complexity of variables associated with oral administration. We compared engraftment of identical preparations and doses of freeze-dried microbiota following colonoscopic (9 patients) versus oral administration (18 patients). Microbiota were characterized by sequencing of the 16S rRNA gene, and engraftment was determined using the SourceTracker algorithm. Oligotyping analysis was done to provide high-resolution patterns of microbiota engraftment. Colonoscopic FMT was associated with greater levels of donor engraftment within days following the procedure (ANOVA *P* = 0.035) and specific increases in the relative abundances of donor *Lachnospiraceae*, *Bacteroidaceae*, and *Porphyromonadaceae* (*P* ≤ 0.033). Lower relative abundances of *Bacteroidaceae*, *Lachnospiraceae*, and *Ruminococcaceae* families were associated with clinical failures. These results suggest that further optimization of oral capsule FMT may improve its engraftment efficiency and clinical efficacy.

## Introduction

Recurrent *Clostridioides difficile* infection (rCDI) is typically a complication of antibiotic disruption of the intestinal microbiota, often compounded by use of successive antimicrobials for treatment of CDI, and represents a major burden on the healthcare system^1–3^. While standard antibiotic treatments suppress toxin-producing vegetative *C. difficile* bacteria, they often fail in eradicating its spores, which germinate in the absence of colonization resistance that is normally provided by the intestinal microbiota and may renew the infection shortly following cessation of the antibiotic^4^.

Fecal microbiota transplantation (FMT) has emerged as an alternative treatment for rCDI and shows ~ 90% clinical success^5^. FMT has evolved over the past decade toward increasingly standardized products that are easy to incorporate into mainstream clinical practice^6^. Cryopreservation of FMT preparations allows for extensive donor selection and testing, which minimizes the potential for adverse events^7–9^. Liquid FMT, frozen with glycerol as a cryopreservative, yields comparable clinical efficacy following colonoscopic and oral capsule administration^10,11^. Recently, we developed an encapsulated, freeze-dried preparation of fecal microbiota for treatment of rCDI that has the advantages of stability and ease of handling and administration^12^. This capsule FMT (cFMT) preparation is approximately 80% effective in curing rCDI and has become the preferred option for the majority of patients in our clinical *C. difficile* program^13^.

Interestingly, normalization of bacterial fecal composition following cFMT is associated with a gradual, punctuated kinetics over a period of a month^13,14^. This relatively slow process contrasts with donor-like normalization of fecal microbial community structure that was seen within a few days after colonoscopic administration of fresh or glycerol-preserved, frozen/thawed suspension of microbiota^15–17^. Notably, the peak incidence of CDI recurrence is the second week following cessation of antibiotics^13,18^. Therefore, the kinetics of donor microbiota engraftment is likely a critical parameter that determines the clinical success of FMT. In fact, we were able to predict clinical failures one week following cFMT in patients with persistently low relative abundances of the bacterial family *Bacteroidaceae* (Bacteroidetes phylum), and the *Lachnospiraceae* and *Ruminococcaceae* families (phylum Firmicutes) in their fecal samples^13^. Similarly, delayed engraftment of donor bacteria contained in SER-109, a commercial bacterial spore-based product, was associated with clinical failure in treatment of rCDI^18^.

The slower engraftment of cFMT relative to frozen liquid preparations may be due to loss of microbial viability following more extensive stress associated with freeze-drying^19^. Alternatively, the slower engraftment of cFMT may be due to a number of variables associated with the oral administration route, such as premature release of capsules in the proximal gastrointestinal tract of some patients, and exposure to the harsh conditions in the stomach and/or small intestine that may compromise survival and engraftment of donor bacteria. Therefore, here we tested the *engraftment potential* of freeze-dried microbiota contained in cFMT capsules by administering the same material colonoscopically. Engraftment was measured in the context of successful clinical treatment of rCDI patients, where FMT was administered colonoscopically or orally using the identical lots and doses of cFMT preparations.

## Results

### Clinical efficacy of capsule FMT (cFMT) and endoscopic (eFMT)

Twenty-seven rCDI patients provided samples for this study, 18 were treated with cFMT and nine were treated with eFMT. The choice of administration route was made by the patients after discussing risks and benefits of both with the treating physician, and all patients were treated with vancomycin prior to FMT by either method. The basic demographics of patients and their clinical disease course were not significantly different (Table 1). Twenty-one percent (4/19) of patients experienced a recurrence of the infection following cFMT, although cure in all patients was achieved following an additional one or more FMT treatments, which were included in subsequent sequence analyses as separate events. The mean time to documented clinical relapse with symptoms and positive stool test for *C. difficile* toxin B PCR was 11 days (range days 3–15). None of the eFMT patients suffered a recurrence of the infection. The difference in the initial clinical outcomes between cFMT and eFMT was not statistically significant.

**Table 1:** Clinical characteristics of patients treated.

	cFMT (n = 18)	eFMT (n = 9)	*P* value
Age, years (mean ± SD)	65 ± 19	60 ± 20	0.5146
Female sex, n (%)	15 (83%)	7 (78%)	> 0.9999
Body Mass Index, kg/m^2^ (mean ± SD)	28.4 ± 10.8	25.9 ± 7.1	0.5347
Median number of months since the initial CDI diagnosis (range)	7.5 (2–36)	5.0 (3–7)	0.0624
History of hospitalization for severe or fulminant CDI, n (%)	6 (32%)	1 (11%)	0.3715
Proton Pump Inhibitor Use, n (%)	3 (17%)	2 (22%)	> 0.9999
Immunosuppression*, n (%)	4 (20%)	7 (18%)	0.2503
Clinical failure, n (%)	4 (20%)	0 (0%)	0.2677

*Immunosuppression is secondary to medications, including prednisone ≥ 20 mg/day, biologics, and/or immunomodulators such as methotrexate or azathioprine. None of the patients in this cohort had inflammatory bowel disease.

### FMT administration route minimally influences bacterial diversity

Changes in both alpha (within-sample) and beta (between-sample) diversity were similar for patients regardless of the route of FMT administration. Alpha diversity, as measured by the Shannon index, was significantly lower among pre-FMT samples (2.9 ± 0.1; Tukey’s *post-hoc P* ≤ 0.001) than was observed among donors (4.5 ± 0.2), or patients who recovered following FMT (3.6 ± 0.1 for both cFMT and eFMT). The route of FMT administration, however, did not significantly affect recovery of alpha diversity after two weeks in both oral and colonoscopy groups. Similarly, the bacterial community composition (Table 2) in patients receiving either cFMT or eFMT differed significantly from that of pre-FMT samples, within the first two weeks following administration (ANOSIM R = 0.173 and 0.214, *P* < 0.0001 for cFMT and eFMT, respectively; Bonferroni-corrected *α* = 0.003). Differences in bacterial community composition between cFMT and eFMT recipients, however, were not significant at either the early (3–14 days post-FMT) or later (15–35 days post-FMT) time points (R = 0.042 and 0.063, *P* = 0.062 and 0.078, respectively). Community composition did not change significantly over time in either group (R = − 0.010 and 0.030, *P* = 0.605 and 0.163, for cFMT and eFMT, respectively).

**Table 2:** Distribution (mean ± standard error, %) of abundant families among sample groups. Families not list†ed each account for a mean < 2.6% of the community, among all samples. Time points (d) reflect days post-FMT. Patients may have collected multiple samples within a given time range.

Family	Donor (n = 3)*	Pre-FMT (n = 28)	Response				Recurrence	
			cFMT		eFMT		cFMT	
			3–14 d (n = 29)	15–35 d (n = 24)	3–14 d (n = 18)	15–35 d (n = 12)	3–14 d (n = 11)	15–35 d (n = 7)
*Lachnospiraceae*	30.1 ± 7.4	8.1 ± 2.1	21.0 ± 1.4^†^	19.5 ± 1.6^†^	24.0 ± 2.3^†^	19.4 ± 2.7	11.9 ± 2.4	13.1 ± 3.7
*Bacteroidaceae*	12.0 ± 2.2	8.1 ± 2.0	14.5 ± 2.3	15.0 ± 3.1	16.4 ± 1.6	14.1 ± 2.1	17.5 ± 3.6	11.0 ± 4.9
*Ruminococcaceae*	19.1 ± 1.9	2.9 ± 1.0	13.2 ± 1.2^†,‡^	12.3 ± 1.6^†^	14.7 ± 1.8^†,‡^	12.9 ± 1.8^†^	4.7 ± 1.5	5.6 ± 1.6
*Verrucomicrobiaceae*	1.2 ± 1.4	3.8 ± 1.5	7.4 ± 1.7	5.6 ± 2.0	7.9 ± 3.2	7.7 ± 3.1	13.4 ± 3.8^†^	17.9 ± 4.6^†^
*Enterobacteriaceae*	0.0 ± 0.0	10.5 ± 1.9	1.1 ± 0.7^†^	4.1 ± 2.5^†^	0.6 ± 0.5^†^	4.8 ± 5.0^†^	3.2 ± 0.8	12.3 ± 3.7
*Porphyromonadaceae*	5.6 ± 3.0^ABC^	7.5 ± 2.2^ABC^	5.9 ± 1.9^ABC^	4.1 ± 1.7^BC^	8.3 ± 1.0^AB^	9.6 ± 1.1^A^	7.5 ± 2.5^ABC^	1.5 ± 1.1^C^
*Rikenellaceae*	2.3 ± 0.6	2.4 ± 1.2	8.0 ± 1.9	8.4 ± 2.2	6.5 ± 1.4	6.3 ± 1.0	4.9 ± 1.8	3.1 ± 1.7
*Erysipelotrichaceae*	2.8 ± 1.3	3.1 ± 0.9	4.2 ± 0.6	3.8 ± 0.6	2.7 ± 0.4	3.2 ± 0.8	5.6 ± 2.4	3.6 ± 2.0
*Sutterellaceae*	2.6 ± 1.0	8.8 ± 1.8	4.8 ± 1.0	6.6 ± 1.5	2.8 ± 0.8	3.1 ± 0.8	4.0 ± 1.0	6.3 ± 2.8
*Lactobacillaceae*	0.0 ± 0.0	8.6 ± 1.9	2.0 ± 1.0	2.1 ± 1.4	0.6 ± 0.6	1.6 ± 1.0	3.6 ± 2.3	5.9 ± 3.3

*Two replicate samples were analyzed for donor 71.

^†^Indicates significant difference from relative abundances in pre-FMT samples (pairwise comparisons using the Steel–Dwass-Critchlow-Fligner procedure, *P* < 0.05). Statistical grouping could not be performed because the significance of differences was not transitive across all groups.

^‡^Indicates significant difference from relative abundances in cFMT recurrence samples collected between 3–14 days (pairwise comparisons using the Steel–Dwass-Critchlow-Fligner procedure, *P* < 0.05). Statistical grouping could not be performed because the significance of differences was not transitive across all groups.

^ABC^Samples sharing the same letter did not differ significantly in pairwise comparisons (*P* > 0.05).

### Administration route impacts engraftment kinetics

We next used the SourceTracker program to evaluate engraftment, defined as the proportion of the bacterial community in patient samples that was attributable to donor communities. Two donors (donors 67 and 71) were used. Donor 67 material from a single production lot was used in eight cFMTs and four eFMTs, while Donor 71 material from a single production lot was used in ten cFMTs and five eFMTs. Overall, the early post-FMT samples from patients who suffered a recurrence of CDI had a significantly lower percent of donor bacteria than post-FMT samples from patients who were cured (46.9 ± 6.8% *vs.* 60.7 ± 3.1%, respectively, *P* = 0.035). Patients who experienced a recurrence also tended to have lower abundances of the predominant families *Lachnospiraceae*, *Bacteroidaceae*, and *Ruminococcaceae*, with greater relative abundances of families that generally only comprised a small proportion of the community (*e.g.*, *Verrucomicrobiaceae*, *Rikenellaceae*, and *Sutterellaceae*), although differences in these associations were not statistically significant (Table 2).

**Figure 1:**
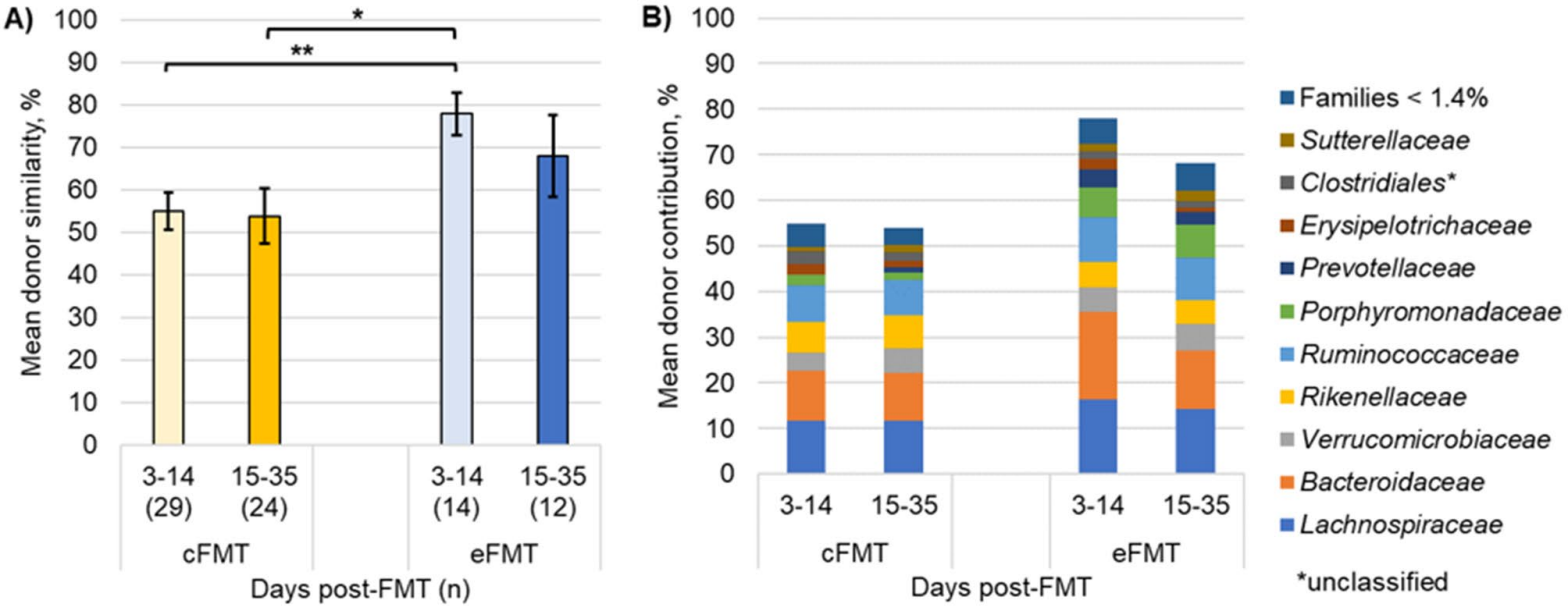
Engraftment of donor fecal bacteria determined using SourceTracker. (**A**) Mean (± standard error) percent of total donor engraftment. (**B**) Family-level distribution of OTUs associated with donor engraftment. Families with a mean < 1.4% of sequence reads among all samples were consolidated. *P < 0.05, **P ≤ 0.01.

Further analyses of engraftment kinetics were restricted only to responders since retreatment with antibiotics impacted the analyses of later time points. We first confirmed that there was no bias in the distribution of patients receiving fecal microbiota preparations from donor 67 or 71 by both routes of administration (*χ*^2^ = 0.000, *P* = 1.000). Eight and ten of the cFMT patients received material from donors 67 and 71, respectively, and four and five eFMT patients received material from donors 67 and 71, respectively (same production lot). Significantly greater engraftment was observed within the first two weeks following eFMT relative to that seen with cFMT (78.0 ± 5.0% *vs.* 55.0 ± 4.3%; Tukey’s *post-hoc P* = 0.002; Fig. 1A), although engraftment levels were similar between both routes of administration after the first two weeks.

### Differential engraftment of bacterial taxa

We then investigated whether specific bacterial families accounted for the greater proportion of engraftment following eFMT. Relative abundances of *Lachnospiraceae*, *Bacteroidaceae*, and *Porphyromonadaceae* were attributed to greater donor similarity using SourceTracker (Kruskal–Wallis *post-hoc P* = 0.041, 0.026, and 0.002, following correction for multiple comparisons; Fig. 1B).

**Figure 2:**
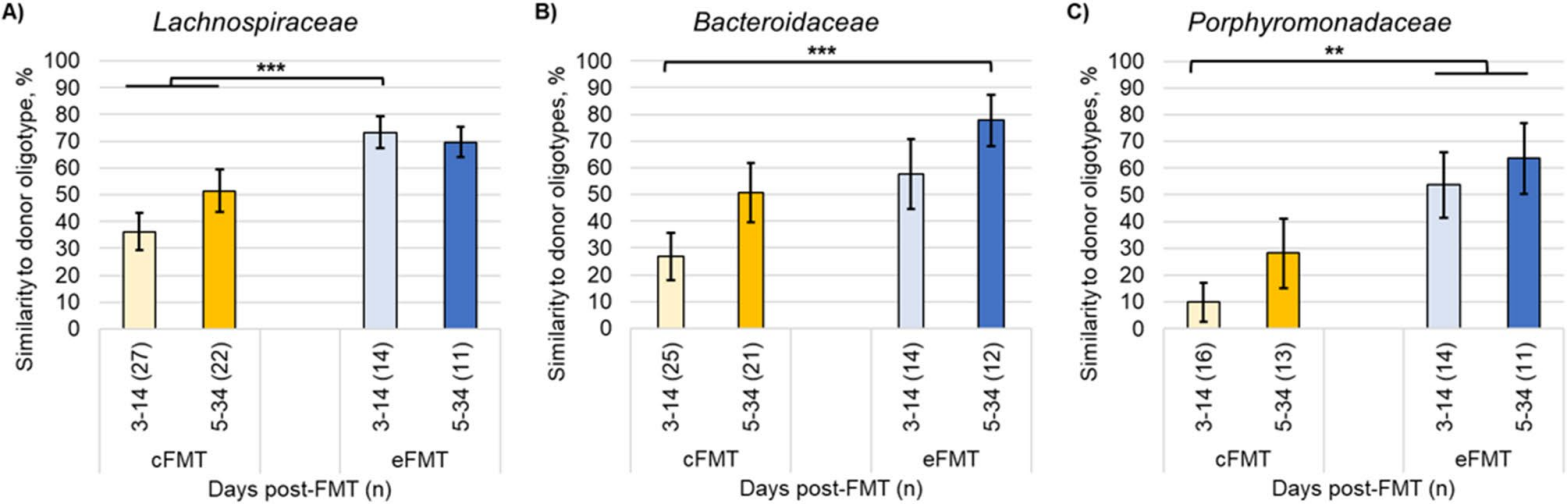
Similarity to donor oligotype profiles. Mean percent similarity to donor oligotype profiles for (**A**) *Lachnospiraceae*, (**B**) *Bacteroidaceae*, and (**C**) *Porphyromonadaceae*, as determined by SourceTracker. Mean was calculated without respect to donor, but only the donor received was used for SourceTracker calculations. Error bars reflect standard error. **P ≤ 0.01 ***P ≤ 0.001.

Oligotyping analyses, which detects unique genotypes based on multiple single nucleotide polymorphisms, were used to better understand diversity among engrafting members of the families *Lachnospiraceae*, *Bacteroidaceae*, and *Porphyromonadaceae*^20^*.* Eighty-two, twelve, and seven oligotypes were observed for the families *Lachnospiraceae*, *Bacteroidaceae*, and *Porphyromonadaceae*, respectively . Among all three families, the oligotype distribution was significantly more similar to that of the donor among patients receiving eFMT than those receiving cFMT (Tukey’s *post-hoc P* = 0.002 and 0.012; 0.033 and < 0.0001; 0.349 and < 0.0001, with respect to bacterial family and donor; Fig. 2). Notably, for all three families, profiles among patients receiving cFMT featured greater abundances of oligotypes that were only detected at low levels in the donor samples. Differences in oligotype distribution did not significantly change after the first two weeks following treatment (*P* ≥ 0.098; Table 3).

**Table 3:** Spearman correlations (*ρ*) between donor oligotype profiles and patient samples.

Family	Donor	Administration	Time point (n)^†^	Average ± SEM
*Lachnospiraceae*	67	cFMT	3–14 days (15)	0.278 ± 0.073 B
		cFMT	15–35 days (8)	0.282 ± 0.130 B
		eFMT	3–14 days (4)	0.483 ± 0.130 AB
		eFMT	15–35 days (3)	0.634 ± 0.013 A
	71	cFMT	3–14 days (12)	0.296 ± 0.062 A
		cFMT	15–35 days (14)	0.290 ± 0.056 A
		eFMT	3–14 days (10)	0.420 ± 0.033 A
		eFMT	15–35 days (8)	0.413 ± 0.050 A
*Bacteroidaceae*	67	cFMT	3–14 days (15)	0.147 ± 0.084 A
		cFMT	15–35 days (8)	0.150 ± 0.122 A
		eFMT	3–14 days (4)	0.324 ± 0.209 A
		eFMT	15–35 days (4)	0.528 ± 0.184 A
	71	cFMT	3–14 days (10)	0.311 ± 0.105 A
		cFMT	15–35 days (13)	0.324 ± 0.093 A
		eFMT	3–14 days (10)	0.722 ± 0.093 B
		eFMT	15–35 days (8)	0.751 ± 0.041 B
*Porphyromonadaceae*	67	cFMT	3–14 days (12)	0.308 ± 0.086 A
		cFMT	15–35 days (4)	0.522 ± 0.157 A
		eFMT	3–14 days (4)	0.445 ± 0.154 A
		eFMT	15–35 days (3)	0.634 ± 0.277 A
	71	cFMT	3–14 days (4)	-0.239 ± 0.146 C
		cFMT	15–35 days (9)	0.108 ± 0.129 BC
		eFMT	3–14 days (10)	0.605 ± 0.207 AB
		eFMT	15–35 days (8)	0.733 ± 0.151 A

^†^Families were not detected in all samples, resulting in variation in the sample sizes among groups.

^A,B,C^Groups sharing the same letter did not differ significantly (Tukey’s *post-hoc P* > 0.05).

## Discussion

Over the last decade, FMT for treatment of rCDI has evolved from infusions of relatively crude preparations of fresh stool to treatments with increasingly standardized preparations. The ultimate goal is the development of highly effective, stable, and simple to administer microbiota-based products. We have achieved ~ 80% cure rate of rCDI with our cFMT product, which contains freeze-dried fecal microbiota, in patients who previously failed multiple antibiotic treatments^13^. However, our pharmacokinetic studies of this preparation demonstrated relatively delayed engraftment of donor microbiota when compared to colonoscopic administration of liquid preparations^12–14^. We hypothesize that delayed normalization of intestinal microbial community structure is a likely explanation for most clinical failures of FMT in treatment of rCDI, and improvement in early engraftment may further enhance FMT cure rates. A similar conclusion was recently reached by investigators of SER-109, a preparation of fecal bacterial spores^18^.

From our perspective, the two likely explanations for delayed engraftment of donor bacteria following cFMT treatment include lyophilization-induced loss of microbiota viability or suboptimal delivery and release of the microbiota to the colon following oral administration. Therefore, this study was done to measure the engraftment potential of freeze-dried microbiota. Colonoscopic administration of the preparation obviates concerns about premature release and transit through the upper gastrointestinal tract. In fact, we did observe that colonoscopic administration of freeze-dried microbiota resulted in faster engraftment of donor bacteria relative to the cFMT using the exact same preparation lots and doses. Furthermore, a greater relative abundance of donor bacteria was seen after a month following eFMT, as compared to cFMT. It is notable that the only clinical failures occurred in the cFMT group, even though the difference was not statistically significant, likely due to the small size of this study.

Consistent with our hypothesis, the non-responders to cFMT had even lesser levels of early donor bacterial engraftment relative to responders receiving cFMT. As a result, similarly to our previous studies^13^, the non-responders had lower relative abundances of the families *Lachnospiraceae*, *Ruminoccaceae,* and *Bacteroidaceae*. The importance of *Bacteroidetes* is consistent with modest superiority of fidaxomicin, which spares Gram-negative bacteria, over vancomycin in treatment of CDI^21^. The roles of *Bacteroidaceae* bacteria mediating colonization resistance against *C. difficile* are not well understood at this time, but potential mechanisms may include facilitation of engraftment of critical members of the *Lachnospiraceae* and *Ruminococcaceae* families, which participate in secondary bile acid metabolism. Secondary bile acids can inhibit *C. difficile* spore germination and vegetative growth, and likely contribute to the mechanisms of FMT in rCDI^22–24^. In fact, increases in fecal concentrations of secondary bile acids correlate with success of FMT^14^. Increases in fecal secondary bile acids were also correlated with cure achieved by SER-109, although failure to achieve desired levels of secondary bile acids in the phase 2 trial was attributed to suboptimal dosing^18^. We do not think that dose is a likely reason for poor engraftment of microbiota in our cFMT preparation because while our standard dose of 5 × 10^11^ cells is more than two-fold greater than the one used in our original studies, we did not see an improvement in cure rate with this adjustment^12^. Furthermore, and anecdotally based on our clinical experience treating ~ 180 rCDI patients with cFMT, we have not observed that higher or multiple doses are clinically superior to single doses in repeat treatments of non-responder rCDI patient with cFMT (data not shown).

Our results strongly suggest that optimization of cFMT administration or its delivery vehicle may improve its clinical success rate. It is possible that the colonoscopy preparation, which results in purging of luminal contents in the colon, increases the ecological ‘space’ for donor microbiota engraftment by decreasing the competitive and physical impact of the indigenous microbiota. It is also possible that cFMT results in premature release of microbiota in some patients due to wide variations in the gastrointestinal transit times. The small size of this study did not allow us to interrogate different host factors that could associate with clinical failure or low donor microbiota engraftment. Future studies should be done to test various administration protocol parameters and host factors, and additional work likely needs to be done to optimize encapsulation design to ensure colonic delivery across the patient population spectrum.

We are aware that this study is limited by its relatively small size. This was not designed to be a randomized, controlled study to test clinical efficacy of cFMT versus eFMT, even though the basic demographics of the patient populations in the two groups were comparable. The goal was to measure the engraftment potential of identical preparations of freeze-dried microbiota delivered in two ways. The levels of engraftment that we observed with cFMT in this patient cohort are consistent with our previous cFMT studies. Here we measured engraftment by using operational taxonomic unit (OTU) designations and evaluated strain-level changes using oligotyping; however, use of metagenomics analysis to achieve better strain level resolution may provide further evidence to explain and optimize differences in engraftment resulting from capsule formulation or administration route. The findings strongly suggest that the outcomes of cFMT with freeze-dried microbiota can be enhanced through systematic research that examines individual variables impacting engraftment. Pharmacokinetic investigations, which in the case of microbiota-based therapeutics include engraftment kinetics, provide important metrics that are likely to correlate with clinical outcomes. Nevertheless, the data from this study can inform power calculations for future investigations.

## Methods

### Healthy donor enrollment and screening

Healthy donors were enrolled according to strict inclusion and exclusion criteria of the University of Minnesota Microbiota Therapeutics Program^7^, and as described in the Investigational New Drug Application 15071. The exclusion criteria included metabolic and autoimmune disorders, any history of gastrointestinal diseases or surgery, allergies, neurologic or psychiatric disorders, or use of antibiotics in the prior six months. Stool used in preparation of every lot of released product was tested for viral, bacterial, and parasitic enteric pathogens, vancomycin-resistant enterococci, methicillin-resistant *Staphylococcus aureus*, carbapenem-resistant *Enterobacteriaceae*, and extended spectrum-beta lactamases. All donor activities including questionnaires, physical examinations, and laboratory testing were approved by the University of Minnesota Institutional Review Board. All procedures and experiments were performed in accordance with relevant guidelines and regulations.

### Freeze-dried microbiota preparation and encapsulation

Encapsulated microbiota was prepared as previously described^12^, using current Good Manufacturing Practices (cGMP) protocols. Briefly, fecal samples were homogenized by blending under N_2_ gas, sieved to remove particles > 0.25 mm, trehalose was added (5% w/w), and freeze-dried. The freeze-dried microbiota was double encapsulated in hypromellose DRcaps from Capsugel (Morristown, NJ, USA) to obtain a final concentration of approximately 1 × 10^11^ cells per capsule. The fraction of bacteria with intact cell membranes was used as an indicator of viability and was determined using a Live/Dead BacLight Bacterial Viability assay kit (Invitrogen/ThermoFisher Scientific, Carlsbad, CA); this fraction did not decrease post-lyophilization relative to fresh microbiota and remained > 50%. Each course of capsules represented only one fecal donation from one of two donors (67 and 71) Capsules were stored at − 80 °C prior to distribution to patients or transport to the endoscopy suite at the University of Minnesota, Minneapolis, MN. The same lots were used for cFMT and endoscopic FMT (eFMT) treatments. Individual healthy donor choice was previously reported to not affect cFMT efficacy^13^.

### Patient enrollment and treatment

Patients with rCDI were offered FMT following at least two prior spontaneous recurrences of CDI despite standard antibiotic therapies with vancomycin or fidaxomicin, and testing positive by PCR for the *C. difficile* toxin within the preceding three months. Recurrence of CDI was defined as a relapse of diarrheal symptoms (≥ 3 loose/watery stool samples for at least two consecutive days) and positive PCR for *C. difficile* toxin gene during a two-month study follow-up^12^. The study was conducted at the University of Minnesota and approved by the University of Minnesota Institutional Review Board. Patients were enrolled over a period of one year (May 2018–June 2019).

The choice of cFMT versus eFMT via colonoscopy was made by the patients following informed consent obtained by the clinical provider that included an extensive discussion of risks and benefits for each route of treatment. In general, eFMT was recommended if there was any diagnostic rationale for performing the colonoscopy, such as concurrent need for colon cancer screening, polyp surveillance, or concern about the potential for underlying inflammatory bowel disease in younger patients. In contrast, cFMT was recommended in the absence of a compelling diagnostic benefit from receiving FMT via colonoscopy. Some patients expressed preference for eFMT because of greater historical track record and reported clinical efficacy. Some patients preferred cFMT because of its relative convenience. As our program does not charge patients or use insurance for the microbiota preparations, their financial burden was limited to costs of colonoscopy and antibiotics that were not covered by medical insurance.

All patients received oral vancomycin prior to FMT, regardless of treatment route. Patients treated with cFMT or eFMT terminated use of vancomycin two or one days prior to treatment, respectively. A colonoscopy preparation consisting of MoviPrep (Salix Pharmaceuticals, Bridgewater, NJ, USA) and 200 mL of magnesium citrate solution was taken only by eFMT patients. No colon purgative was given to cFMT patients. Omission of such preparations, which are burdensome to many rCDI patients who are often elderly and fragile, in our early cFMT experiences still allowed for high clinical efficacy^12^. Encapsulated cFMT preparations were home-delivered by a research coordinator and taken on an empty stomach with only clear liquids for the following two hours. A single treatment dose consisted of four capsules. The same encapsulated preparation was used for colonoscopic delivery; the capsules were manually opened and hydrated in 120 mL sterile normal saline prior to application. The suspension was administered into the terminal ileum or cecum during colonoscopy. Both delivery methods provided a dose at 5 × 10^11^ bacteria.

### Sample collection and processing

Fecal samples were collected in single-use toilet hats. An aliquot (scoop) of stool was then transferred by the patient into a 30 ml polystyrene fecal specimen container (Globe Scientific, Inc., Paramus, NJ, USA). Samples were frozen in the patients’ home freezers, transferred to the lab on ice, and stored at − 80 °C until DNA extraction. Samples were collected prior to treatment and at approximately 1-, 2-, and 4-weeks following treatment. In correspondence with our previous studies^13,14,25^, samples were grouped in two-week intervals (*i.e.*, 3–14 days and 15–35 days post-IMT). In some cases, patients collected multiple samples within these time windows, and two replicate samples were collected and analyzed for donor 71. DNA was extracted from thawed fecal samples (approximately 250 mg) using the DNeasy PowerSoil Kit (QIAGEN, Hilden, Germany) on the automated QIAcube platform using the inhibitor removal technology (IRT) protocol.

### Sequencing

The V5-V6 hypervariable regions of the 16S rRNA gene were amplified using the BSF784/1064R primer set^26^. Dual-index, paired-end sequencing was done on the Illumina MiSeq platform (Illumina Inc., San Diego, CA, USA) at a read length of 300 nucleotides. Amplification and sequencing were done by the University of Minnesota Genomics Center (Minneapolis, MN, USA), as previously described^27^. Raw sequence data are deposited in the Sequence Read Archive^28^ under BioProject accession number SRP070464.

### Bioinformatics

Sequence data were processed using mothur version 1.41.1^29^ and our previously published pipeline^13^. Briefly, samples were paired-end joined, screened for quality, and aligned against the SILVA database (version 132)^30^. A 2% pre-cluster and UCHIME version 4.2.40 were used to remove sequence errors and chimeras, respectively^20,31^. Operational taxonomic units (OTUs) were binned at 97% sequence similarity using the furthest-neighbor algorithm, and taxonomic annotations were made against the version 16 release from the Ribosomal Database Project^32^.

Analysis of engraftment was done using SourceTracker version 0.9.8 and default settings^33^. This algorithm uses a Bayesian inference model to attribute OTUs from the user-defined source (donor) samples to sink (patient) samples. We previously demonstrated that this tool provides a conservative measure of bacterial engraftment^25^. For all analyses, patients were grouped separately based on the donor they received.

Oligotyping was done using mothur2oligo version 2.1 software and recommended best practices^34^. This analysis evaluates entropy (variability) across each nucleotide position and uses these highly variable single-nucleotide polymorphisms to achieve strain-level resolutions. Oligotyping analysis on members of the families *Lachnospiraceae*, *Bacteroidaceae*, and *Porphyromonadaceae* were done based on observed differences in engraftment. The parameters -M 100 -a 1 -A 500 -s 10 were used for all three families, specifying that a unique oligotype represented at least 1% of sequence reads, was detected in at least 10 samples, and had minimum of 100 unique reads. Eighteen entropy positions were used for *Bacteroidaceae* and 22 were used for *Lachnospiraceae* and *Porphyromonadaceae*, based on best practices to optimize the number of oligotypes identified.

### Statistical analyses

Alpha and beta diversity statistics were calculated in mothur following rarefaction to 11,000 reads per sample for unbiased comparisons^35^. The Shannon index^36^, accounting for richness and evenness, was used to evaluate alpha diversity. Beta diversity was evaluated based on the Bray–Curtis distances^37^. Beta diversity was visualized by ordination via non-metric multidimensional scaling^38^. Differences in community composition were evaluated using analysis of similarity (ANOSIM)^39^, with Bonferroni correction for multiple comparisons.

Differences in clinical response and effects of administration route and donor were evaluated using the *χ*^2^ test. Differences in Shannon index, SourceTracker engraftment, and oligotype correlation coefficients were determined using ANOVA with Tukey’s *post-hoc* test. Differences in taxon abundances were evaluated using the non-parametric Kruskal Wallis test, using the Steel–Dwass–Critchlow–Fligner procedure for pairwise comparisons. Correlations between oligotype profiles were determined by Spearman correlation analyses. Statistical analyses were performed using XLSTAT software version 2020.3.1 (Addinsoft, Belmont, MA).
